# Association of liver X receptor α (LXRα) gene polymorphism and coronary heart disease, serum lipids and glucose levels

**DOI:** 10.1186/1476-511X-13-34

**Published:** 2014-02-17

**Authors:** Yun-Fei Zhou, Jing Zhang, Zong-Xue Li, Jing-Li Miao, Qiao-Xiang Yin, Jun-JIE Li, Xiao-Yan Zhang, Yuan-Yuan Li, Hui-Lan Luo

**Affiliations:** 1 The cadre ward of General Hospital of the Air Force PLA, No. 30, Fucheng Road, Haidian District, Beijing 100142, China; 2 Combination of traditional Chinese and Western Medicine Hospital of Southern Medical University, Guangzhou 510515, China

**Keywords:** Liver X receptor α, Gene, Coronary heart disease, Polymorphism

## Abstract

**Background:**

To explore the relationship between the liver X receptor α gene (LXRα) rsl2221497 polymorphism and the susceptibility of coronary heart disease (CHD) and serum lipids and glucose levels.

**Methods:**

The single fluorescently labeled probes technique was used to detect the genotype of rsl2221497 in LXRα gene in 240 CHD patients and 250 healthy control subjects. The difference of genotype distribution between the two groups was analyzed using of Chi-square test. The serum lipids and glucose levels between the different genotypes were also compared.

**Results:**

The risk of CHD in carriers with (AA + GA) genotype was 1.76 times as that in the GG genotype carriers (OR = 1.76, 95% CI: 1.18-2.87, *P* <0.05), and the risk of CHD in carriers with A allele increased 0.88 times compared to that in G allele carriers (OR = 1.88, 95% CI:1.21-3.43, P <0.01). Logistic regression analysis showed that after adjusting for other confounding factors, A allele was an independent risk for CHD. However, there were no differences in serum lipids and glucose levels between each genotype.

**Conclusions:**

The rsl2221497 polymorphism in LXRα gene was associated with susceptibility of CHD in Han population.

## Background

Coronary heart disease (CHD) is a multifactorial disease resulting from the interaction between genetic factors and environmental factors [1-4]. Currently, it is considered that vascular endothelial cell injury, platelet reactivity, lipid infiltration, vascular smooth muscle cell proliferation and increased synthesis of connective tissue are the main pathological processes of CHD [5,6]. Lipid metabolism disorder, especially elevated serum LDL-C is an independent risk factor for CHD [7]. Liver X receptor α (LXRα) is a class of nuclear receptor family members [8]. These members are mainly distributed in the liver, adipose tissue, kidney, small intestine and macrophages, and the main physiological function is to control stable internal environment of cholesterol level, lipoprotein metabolism and fat synthesis [9,10]. The current pathophysiological role of LXR α in humans is not entirely understood, but some researchers identified this gene as a candidate gene of metabolic syndrome and diabetes [11,12]. Diabetes is equivalent risk of CHD, however, there was still no research data on the relation between gene polymorphism of LXRα and CHD. In the present study, we performed a case - control study to analyze the association of LXRα gene polymorphism with CHD in a Chinese Han population.

## Methods

### Ethnics

The present study has been performed with the approval of the ethics committee of General Hospital of the Air Force PLA and is in compliance with the Helsinki Declaration. The informed consents of the study were collected from all the candidate subjects.

### Subjects

From August, 2009 to June, 2012, two hundred and forty hospitalized patients diagnosed as CHD in our hospital were enrolled as the case group, in which one hundred and ninety-one patients were male and forty-nine patients were female. The average age was 61.22 ± 10.22 years. CHD was diagnosed according to WHO issued ischemic heart disease diagnostic criteria in 1979. In the light of coronary angiography, ≥ 50% of stenosis of the main branches was defined as CHD.

In control group, 250 healthy inpatients were selected during the same period in our hospital, 192 were male and 58 were female with mean age of 61.43 ± 11.21 years. The disease history, physical examination, laboratory tests (serum, urine, stool routine tests, serum lipids and glucose), ECG and other preliminary tests were performed. The history of CHD, hypertension and diabetes were detected. All the subjects were Han Chinese people.

### Clinical data collection

The clinical data including past medical history, family disease history, smoking and drinking history, weight, height, blood pressure, body mass index (BMI) were collected in the present study.

2 mL of 12 h-fasting venous blood was collected, and the triglyceride (TG), total cholesterol (TC), high density lipoprotein cholesterol (HDL-C), low density lipoprotein cholesterol (LDL-C), fasting blood glucose (FBG) and other biochemical parameters were detected using biochemical analyzer (NJ, USA).

### Preparation of DNA from peripheral blood leukocytes

100 uL of EDTA anti-coagulated blood was taken, and the blood genomic DNA was extracted from whole blood according to the protocol of DNA-extraction kit (Shanghai Shennengbocai company) and was dissolved in TE solution and stored at -20°C refrigerator.

### Instrument and reagents

Light Cycler gene amplification detector was utilized for PCR reaction. Taq DNA polymerase, 4 × dNTPs, 10 × Bufier buffer and MgCl_2_ were purchased from Shanghai Shennengbocai company (Shanghai, China). Primer sequence was as follows: upstream primer: 5 '-GGCTTACTC CAA TAA TCC CCA CAC TT-3’, downstream primer: 5'-AAGGAAGAAGGCAGGTAATGATGAAGGAG-3' (synthesized and modified by Shanghai Sangon Biotechnology Co., Ltd.).

### Statistical analysis

Statistical analysis was performed using SPSSl3.0 (IL, USA). Continuous data were expressed as mean ± standard deviation (M ± SD). Difference between the two groups was compared by using of *t* test. Genotype and allele frequency distribution in the case group and the control group were calculated with direct counting method. Hardy-Weinberg equilibrium was performed using Chi-square test, genotype and allele frequencies distribution between the two groups were compared using chi-square test or Fisher's exact test. Multivariate Logistic regression model was used to analyze risk factors of CHD, the odds ratios (OR) and 95% confidence intervals (95% CI) were used to express the relative risk.

## Results

### Genotyping results

Three genotypes were detected in rsl2221497, namely as GG homozygote, AA homozygote, GA heterozygote.

### General clinical data comparison in the control group and the CHD group

The mean age, sex and body mass index (BMI) were not significantly different between the two groups (*P* > 0.05). However, there were significant difference between the two group in TG, TC, HDL-C, LDL-C, FBG (all *P* <0.05, Table 1).

**Table 1:** Demographic and risk profile of the study population

**Risk factors**	**No. (%) or Mean ± SD**		**P values**
	**Control (n = 250)**	**CHD (n = 240)**	
Age (years)	61.43 ± 11.21	61.22 ± 10.22	0.332
Female (%)	58 (24.2)	49 (19.6)	0.130
BMI (Kg/m^2^)	24.52 ± 3.40	24.76 ± 3.91	0.101
GLU (mmol/L)	4.20 ± 0.55	5.27 ± 0.75	<0.001
TG (mmol/L)	1.36 ± 0.64	2.13 ± 0.44	<0.001
TC (mmol/L)	4.29 ± 0.79	5.12 ± 0.87	0.005
HDL-C (mmol/L)	1.29 ± 0.49	1.01 ±0.44	0.043
LDL-C (mmol/L)	2.26 ± 0.80	2.89 ±0.81	0.038
Smoking (n)	97	132	0.011
Hypertension (n)	82	144	0.002
Diabetes (n)	44	87	0.016
Obesity (n)	68	79	0.077

Note: HDL, high-density lipoprotein; LDL, low-density lipoprotein; TG: Triglycerides; TC: Cholesterol; BMI: Body mass index; GLU: Glucose.

### Genotype and allele frequency distribution

Genotypes in CHD and the control group were in line with Hardy-Weinberg equilibrium (*P* > 0.05) indicating that the genotype frequency distribution reached the genetic equilibrium, the sample can represent the whole group. Genotype analysis showed that the CHD risk in AA + GA genotype carriers was1.76 times as that in GG genotype carriers (OR = 1.76, 95% CI: 1.18-2.87, *P* <0.05). A allele frequency in CHD group was significantly higher than that in control group (*P* <0.01), and the odds ratio (OR) was 1.88 (95% CI:1.21-3.43), Table 2.

**Table 2:** Distributions of SNPs of LXR gene in case and control group

**rsl2221497**	**Genotypes**	**Group**		**P value**
		**Control, n (%)**	**CHD, n (%)**	
Genotype	GG	215 (86.0)	185 (77.1)	0.012
	GA	33 (13.2)	48 (20.0)	
	AA	2 (0.8)	7 (2.9)	
Allele	G	463 (92.6)	425 (87.1)	0.004
	A	37 (7.4)	55 (12.9)	

### Serum lipids and fasting glucose in different genotypes

There were a total of 490 cases in coronary heart disease and the control group, 400 cases had GG genotype and 90 cases had GA + AA genotype. The difference of TG, TC, HDL-C, LD-C, FBG between the different genotypes was not shown statistically significant (P > 0.05), Table 3.

**Table 3:** Serum lipids and glucose levels between each genotype

**Lipids and glucose**	**No. (%) or Mean ± SD**		**P values**
	**GG (n = 400)**	**GA + AA (n = 90)**	
GLU (mmol/L)	4.88 ± 0.76	4.97 ± 0.64	0.432
TG (mmol/L)	1.75 ± 0.68	1.78 ± 0.49	0.654
TC (mmol/L)	4.58 ± 0.87	4.69 ± 0.66	0.179
HDL-C (mmol/L)	1.14 ± 0.33	1.10 ±0.43	0.186
LDL-C (mmol/L)	2.47 ± 0.88	2.64 ±0.75	0.078

Note: HDL, high-density lipoprotein; LDL, low-density lipoprotein; TG: Triglycerides; TC: Cholesterol; BMI: Body mass index; GLU: Glucose.

### Logistic regression analysis

In multivariate Logistic regression analysis, the CHD was taken as the dependent variable. The age, sex, BMI, TC, TG, LDL-C, HDL-C, FBG, A allele, smoking history, hypertension history, diabetes mellitus history were taken as independent variables in Logistic regression analysis. The results showed that HDL-C, TC, TG, history of hypertension, smoking history, age, BMI, A allele were independent risk factors for CHD, the OR for A allele was 1.85 (*P* <0.05, 95% CI: 1.09 ~ 3.55) after adjustment of other confounders.

## Discussion

In this study, we found that in Han population, LXRα A allele increased the risk of CHD. This result was consistent with LXRα physiological role in the human body. LXRα can regulate many target genes involved in lipid uptake, spillover and lipid metabolism [13]. The regulation function of activated LXR α was as follows: 1) it can mediate the binding and transporting factor Al (ABCAl), ABCGl, ABCG5, ABCG4, ABCG8 located in the human macrophages and small intestine target genes ATP to promote endogenous lipid membrane transport; 2) it can activate human macrophages Niemann - Pick Cl protein (NPCI) and C2 protein (NPC2) to promote cholesterol transport from endosomes chamber to the cytoplasmic membrane; 3) it can promote receptor ApoE, ApoC-I, C-II, C-IV expression which were in charge of regulating the cholesterol outflow in adipocytes and macrophages; 4) it can control the liver and macrophages regulating enzymes such as phospholipid transfer protein (PLTP), lipoprotein lipase (LPL) remodeling lipoproteins. Meanwhile LXR α can inhibit many inflammatory cytokines and the expression of chemokines [14-18]. All these indicated that LXR α signaling pathway played an important role in the development of atherosclerosis. The mice tests also proved this view, the synthesis of liver X receptor agonist can inhibit the development of atherosclerosis, and the effects may be due to regulation of the underlying metabolic and inflammatory gene expression [19,20].

Our studies suggested that in Chinese Han patients with CHD, the LXRα A allele frequency was significantly higher than that in the healthy population, A allele carries had 0.8 times increased risk of CHD (OR = 1.81). In the multivariate Logistic regression analysis, after the adjustment of age, sex, cholesterol, fasting glucose, hypertension, diabetes, smoking history and other confounding factors, A allele was still a risk factor of CHD independent of other traditional risk factors (*P* <0.05) which indicated that this locus had significantly increased risk for CHD. A recent study indicated that in the female population, LXR α polymorphism was significantly correlated with BMI and HDL-C concentration [21]. In a French-Canadians population, the serum cholesterol levels in A allele carriers were higher than those in GG homozygotes carriers [22]. The dietary cholesterol intake and this locus polymorphism had the combined effects on plasma TC and LDL-C levels suggesting that the plasma lipoprotein concentrations were not only associated with dietary cholesterol, but also regulated by LXR α gene expression. This locus was in recognition area of LXRα gene transcription factor and was involved in LXRα transcriptional regulation. In the present study, we did not find significant differences in lipids and BMI between A allele carriers and GG homozygotes carriers. This suggested that there may exist heterogeneity between different ethnics and populations, the diet and racial and body size differences between Han population and Caucasian may also be an explanation.

There were several limitations in our study. Firstly, the relative small sample size may reduce the statistics power and overestimate the OR value. Secondly, in the present study, we only investigated one SNP in LXRα gene. Although many previous studies suggested this SNP was associated risk factors of CHD, this SNP may not figure out the relationship between LXRα polymorphism and serum lipids and CHD risk. Finally, we only investigate this association in one case-control study, another independent case-control study for verification was not designed.

## Conclusion

In conclusion, this study first reported the association between genetic variation of LXR α gene and CHD in Han population. It provided some new clues to further understand the mechanism of the development of CHD and prevention. The study also confirmed from the genetic level that the lipid metabolism was the risk factor of CHD.

## Competing interests

The authors declared no competing interests exist.

## Authors’ contributions

YFZ and ZXL carried out the molecular genetic studies and drafted the manuscript. YFZ and JZ carried out the genotyping. QX, JJL, and XYZ participated in the design of the study and performed the statistical analysis. YYL conceived of the study, and participated in its design and coordination and helped to draft the manuscript. All authors read and approved the final manuscript.
